# Self-esteem, peer pressure, and demographic predictors of attitude toward premarital sexual practice among first-year students of Woldia University: Implications for psychosocial intervention

**DOI:** 10.3389/fpsyg.2022.923639

**Published:** 2022-08-17

**Authors:** Wossen Ayalew Tegegne

**Affiliations:** College of Education and Behavioral Sciences, Bahir Dar University, Bahir Dar, Ethiopia

**Keywords:** premarital sex, self-esteem, peer pressure, demographic variable, university students, attitude

## Abstract

Adolescence is s period of storm and stress mainly caused by dramatic social, emotional, biological, and psychological changes. Unless supported and guided by parents, teachers, peers, and other stakeholders, they may be exposed to a variety of risky sexual behaviors. In the study area, there is no research work showing the influences of peers, self-esteem, and demographic factors on the premarital sexual practice among students. Ideas and knowledge gained in this area will enable the delivery of effective health and psychosocial intervention strategies. Thus, the objectives of this research were to assess self-esteem, peer pressure, and demographic predictors of attitude toward premarital sex among Woldia University first-year students. Correlational design was used and 343 students were selected as samples by a simple random sampling method. Relevant information was gathered using a questionnaire. The data were quantitatively analyzed using logistic regression, one sample, and an independent sample *t*-test. This research work showed that level and exposure to peer pressure among first-year university students having the experience of premarital sex have below-average levels of self-esteem (*t* = −40.93, *p* = 0.000) and have been exposed to a higher level of peer pressure (*t* = 23.27, *p* = 0.000). The study suggests that male first-year students with the experience of premarital sex have a higher level of self-esteem and exposure to peer pressure than their female counterparts. Self-esteem negatively predicted attitude toward premarital sex. Therefore, the researcher recommended delivering effective counseling and gender-based guidance, and life skill training for fresh students in the university.

## Introduction

The age of adolescence is a move from late childhood to early adulthood, characterized by rapid bodily, psychological, emotional, and biological changes. Even if they have matured sexual organs, their mood and personality swings. This fluctuation of mood and personality can affect their social relationship and their attempt to identity formation. Thus, adolescents should perform adult-like activity carefully in order to develop their identity (Malhotra, 2008). More than any other time, currently adolescents’ sexual behavior is troubling. 40 and 75% of adolescents experience premarital sex between the age of 15–19 and 20–24 years, respectively. In relation to this, each year nearly, nine million new cases of sexually transmitted diseases occur at ages ranging from 15 to 24 (Richards, 2013).

The prevalence of the premarital sex in both developed and developing countries is high and our country was a victim of such practice (Abiy et al., 2018). Today, many adolescents practice sexual intercourse at 18°years. On the basis of the survey, 50% of the grade, 9th and 12th students conveyed that they have premarital sex. The probability of engaging in premarital sex arises when age increases, nearly by grade, 12th almost two-thirds of students have had sex (Robert et al., 2002).

Most of the students at the age of late adolescents did not agree with indulging in premarital sex. Mass media were the main sources of sexual information for men and women learned through school or peer groups. Emotional factors such as self-confidence, love, trust, fear, conformity, self-esteem, and jealousy cause premarital sex among adolescents. Lack of parental support and supervision, contraception, more women in the workforce, and use the decline of marriage are also other reasons for the commitment to premarital sex (Srishti and Ayesha, 2018).

The engagement in premarital sexual exercises extended the self-perceived worth oneself. Self-esteem gets to be higher when abstaining from sexual intercut some time recently conjugal (Jones, 2010). There is a relationship between self-esteem with premarital sexual behavior: Higher self-esteem diminishes premarital sexual behavior. It is related to premarital sexual behavior in adolescents of 24.4% (Winarni et al., 2016). Gentzler and Kerns (2004) also found that women having high self-esteem had more sexual partners than those with fewer sexual partners. Self-esteem has a direct influence on premarital sex. In this respect, in their study, Weaver and Herold (2000) noted that 25% of women who practiced premarital sex expressed that they did so to upturn their self-esteem and 24% of women who had not intercourse assumed that they would have improved self-esteem if they participate in casual sex.

Peers are a group of individuals in the same developmental period, and with communal behaviors and emotions, who can demonstrate faithfulness and a feeling of fitting into the group. In adolescence, peers are the young people who spend time with and interact with them. In this stage, adolescents devote more of their time and communicate in a better way with their peers than in other stages of development (Liu, 2005). Peers’ popularity and conformity, peers’ influence, and the need to be famous and accepted are contributing factors to why adolescents spend their time with friends. Abusive behavior, which reflects the supremacy, of men toward women is a kind of peer culture (Clasen and Brown, 2000).

Undesirable peer pressure is stronger for women than men about sexual behavior. Peer pressure may be both expressed and indirect. In expressing peer pressure, an individual is confronted openly to conform to the prevailing values, norms, and cultures in general. Research findings indicated that both peers are tending to take risks they do not want to take because they believe that this risky behavior will increase their standing and relation in the eyes of their peers and guarantee their acceptance in their group (Cotterell, 2007).

Peer pressure substitutes the impact on adults and became the source of information, values, and behavioral influences for adolescents over the last 50 years (Neufeld and Mate, 2005). Today, the participation of peers in antisocial behavior increased dramatically. The negative actions of one member of a group will increase the possibility of other members taking part in similar behavior more powerfully than community, school, or family characteristics (Gifford et al., 2005).

Researchers showed that men are more likely to start sexual intercut and have more tolerance to recognize approximately sex than women (XiayunZuo et al., 2012). Male and female students have almost the same rate of permissive attitudes toward premarital sex but men are more permissive (Ifeoma, 2014).

Attitude toward premarital sex is a potent factor in the premarital sexual relationships. Adolescents’ attitude toward premarital sex, to a great extent, determines their behavior regarding sexuality *vis-à-vis* becoming sexually active or becoming abstinent. Adolescents in junior and senior schools display a positive attitude toward premarital sex as the majority of them do not see that going without sex would make them wiped out or see odd in society, not one or the other would it deliver them issues amid intercut when they at long last marry (Tsotovor and Dadey, 2021). Premarital sex is appropriate to satisfy the sexual desire of an individual. Because of this, more than half of both men and women have a positive attitude toward premarital sex (Bhatta et al., 2016).

Studies conducted in some towns of Ethiopia among high school students showed the prevalence of the premarital sexual practice. In this respect, in Goba town, 31.16% (Shemsu and Tilahun, 2020), Sebeta town, 28.3% (Berihun, 2014), Bahir-Dar, 30.8% (Mulugeta and Berhane, 2014), Maichew, 29.3% (Salih et al., 2015) Gondar and Metema, 31.9% (Habte et al., 2017), and Agaro, 25% (Girma et al., 2004) of participants of high school students had premarital sex. On the other hand, a study conducted at Wollega University, Ethiopia indicated that 28.4% of the undergraduate regular students of the university were engaged in premarital sexual practices. Being man, frequent utilization of social media, and alcohol consumption was associated with the premarital sex (Tesfaye et al., 2016).

On the contrary, a study at the Madawalabu University, Ethiopia demonstrated that 181 (59.9%) respondents who had male or youthful female companions; around 129 (42.7%) have had premarital sexual interdenominational. Out of sexually energetic respondents, 85 (66.4%) had one sexual accessory, and 44 (33.6%) had two or more sexual accessories. The typical age for starting sexual intercourse was 18.4 ± 2.14 years (Tomas et al., 2017).

Different studies suggested that demographic factors such as sex, age, educational background, drinking alcohol, substance use, and lack of parental support and supervision were linked with premarital sexual activity (Abiy et al., 2018). Even though the magnitude of the problem in Ethiopia is expected to be high, there was a shortage of studies regarding premarital sexual practice, particularly at the current study site where many young lived. Therefore, this study aimed to assess self-esteem, peer pressure, and demographic predictors (only sex) of attitude toward premarital sexual practice among students of Woldia University. Thus, this research was designed to answer the basic research questions listed later.

1. What is the level of self-esteem and exposure to peer pressure among students who have experience of premarital sex?

2. Are there sex differences in the level of self-esteem and exposure to peer pressure among students having an experience premarital sex?

3. To what extent do sex, self-esteem, and peer pressure variables predict attitude toward premarital sex?

## Materials and methods

The correlational research design was employed to investigate this study. The target populations of this study were all first-year students of Woldia University, and there are 2,424 first-year students exist among those 178 males and 165 female students. A total of 343 students were selected as a sample of this study by using simple random sampling techniques. The researcher takes the sample only from first-year students purposively because; during the study, they were only admitted to attend their class because of COVID-19 protocol. The participants of this research were determined by using Yamane (1967) formula:


$$n=\frac{N}{1+N\,\left(\mathrm{e2}\right)}$$

where; *n* = is the sample size, *N* = is the total population, 1 = is constant and *e* is the compromise margin of error, (in social science there is a 95% confidence level, and *e* = 0.05).


$$n=\frac{2424}{1+2424\,\left(0.0025\right)}=343.$$

So, the sample size of this study was 343 students.

A questionnaire was used to collect information regarding attitudes toward peer pressure, self-esteem, and demographic characteristics of the participants toward the premarital sexual practice of university students. The questionnaires and aim of the study were limited and prepared to measure demographic variables (sex and age) and psychosocial factors of self-esteem and peer pressure. In total, 80 students were selected and a pilot test was conducted among the students of the Debre Tabor University.

Perception of Peer Pressure Scale developed by Manzoni et al. (2011) was adapted and used for this research. Respondents were told to rate from 18 Likert Scale questions ranging from “0-Not true at all” to “3-completely true,” with higher scores indicating the prevalence of greater peer influence. As reported by Manzoni et al. (2011), the internal consistency of the scale was 0.83.

On the contrary, the self-esteem measuring scale developed by Rosenberg (1965) was adapted and employed for this study. The tool is a four-point Likert scale having 22 items (ranging from strongly agree to strongly disagree) which allowed participants to rate their responses. The reliability of the self-esteem scale was 0.77.

On the contrary, the sexual attitude inventory scale which was developed by Eysenck and Eysenck (1971) was adapted and used as the main source of data collection for the present study. In total, 24 items were used to measure the intended behavior of the samples. The internal consistency of this scale was 0.97.

First, a cooperation letter was taken from the college and submitted to the concerned body. Detail orientations about the objectives of the study were given to participants of the study and assistant data collectors. Participants were also told that the info they provide will be kept confidential and the right of withdrawal. After the researcher gained written consent from participants, data were gathered in the face-to-face fashion. In total, four competent assistant data collectors were employed to gather relevant data from participants.

All the procedures of the research were supervised by the research and community service coordinating office of the faculty. Subsequently, the collected data were analyzed by using mixed methods of analysis, i.e., quantitative and qualitative methods. Percentage, independent sample *t*-tests, and binary logistic regression were used to investigate numerical data. On the contrary, phrases, words, and sentences were employed to examine qualitative data. Tables were used to present the data. The collected data were analyzed by using SPSS version 20.

## Results

### Background

In total 178 (51.89%) of the respondents were men and the remaining 165 (48.11%) were women. The majority of the participants were aged 18–20°years, and all the respondents were never married in their lifetime and the majority of them have one sexual partner and friend (Table 1).

**TABLE 1:** Background of respondents.

Characteristics		Frequency	%
Sex	Male	178	51.89
	Female	165	48.11
Age	18	78	22.74
	19	188	54.81
	20	62	18.08
	21	15	4.37
Marital status	Never married	343	100
Age at sexual debut	18	98	28.58
	19	176	51.31
	20	56	16.33
	21	13	3.79
Sexual partner	Acquaintance	125	36.44
	Friend	218	63.56
Partner number	1	310	90.38
	2	33	9.62

### Level of self-esteem and exposure to peer pressure

The level and exposure to peer pressure among first-year university students having the experience of premarital sex have a below-average level of self-esteem (*t* = −40.93, *p* = 0.000) and have been exposed to a higher level of peer pressure (*t* = 23.27, *p* = 0.000) (Table 2).

**TABLE 2:** Level of self-esteem and exposure to peer pressure among students having an experience of premarital sex.

Variables	Observed mean	SD	Test value	Df	*T*	Sig. (2-tailed)
Self Esteem	15.30	2.77	22.5	342	−40.93	0.000
Peer Pressure	20.73	3.33	20	342	23.27	0.000

### Sex differences in the level of self-esteem and exposure to peer pressure

As presented in the earlier table, there is a significant difference in self-esteem among men (mean = 16.86, SD = 2.76) and women (mean = 15.78, SD = 2.04), *t* = 3.97, *p* = 0.000. In a similar manner, significant difference in exposure to peer pressure between men (mean = 28.05, SD = 4.75) and women (mean = 25.16, SD = 3.18), *t* = 5.82, *p* = 0.000. This shows that male students in the first year with the experience of premarital sex have a higher level of self-esteem and exposure to peer pressure than their women counterpart (Table 3).

**TABLE 3:** Sex differences in the level of self-esteem and exposure to peer pressure among students having the experience of premarital sex.

Variables	Sex	Mean	Sd	*T*	
Self Esteem	Male	16.86	2.76	3.97	0.000
	Female	15.78	2.04		
Peer Pressure	Male	28.05	4.75	5.82	0.000
	Female	25.16	3.18		

### Attitude toward premarital sex on sex, self-esteem, and peer pressure

The full model containing the three predictors (sex, self-esteem, and peer pressure) was not statistically significant χ^2^ (3, *N* = 343) = 8.836, *p* = 0.03. This model elucidated that only 3.2% (Cox & Snell *R*^2^) and 4.7% (Nagelkerke *R*^2^) of the variance in attitude toward premarital sex. The model also classified 78.7% of the cases correctly.

It further shows that only self-esteem emerged as a unique significant contributor to the model with an odds ratio of 0.878. This suggests for every unit of growth in self-esteem respondents are 0.63 times less likely to have a favorable attitude toward premarital sex. This shows self-esteem negatively predicts attitude toward premarital sex (Table 4).

**TABLE 4:** Regression of attitude toward premarital sex on sex, self-esteem, and peer pressure.

Variables	*B*	S.E.	Wald	Df	Sig.	Exp (B)
Sex	–0.132	0.326	0.185	1	0.691	0.878
Self-esteem	–0.141	0.062	5.312	1	0.021	0.867
Peer Pressure	0.013	0.037	0.091	1	0.763	1.011
Constant	3.176	1.656	3.679	1	0.055	23.949

## Discussion

The study indicates that students having the experience of premarital sex have below-average levels of self-esteem. This result was related to Geckil and Dundar (2011). Their study showed that adolescents who scored low on self-esteem had a higher tendency to be exposed to premarital sexual behaviors. On the contrary, this result is also consistent with the work of Lejuez et al. (2004) who were arguing that low self-esteem causes individuals to practice risky sexual behaviors such as unsafe sex, and having unlimited sexual spouses and sex before marriage. In a similar manner, the work of Bhana and Lombard (2004) also validates that low self-esteem is strongly associated with unsafe sexual behavior and premarital sex among adults.

In a similar manner, this discovery additionally supports these previous research (Hidayat, 2013; Aditomo and Retnowati, 2014; Winarni et al., 2016 among others), which confirmed that there is a relationship between self-esteem and premarital sexual behavior. Self-esteem is greater when abstaining from sexual intercourse before marriage. Individuals who have low self-esteem are terrible and will normally feel less satisfied, less capable, and much less valuable than others.

This study also shows that university student having the experience of premarital sex has been exposed to a higher level of peer pressure. This result aligns with the results of Anyama (2019) who explained that same-sex peers were the main source of sexual-related information and peers also afford setting where sexual activities take place. By taking a sexually experienced friend as a role model, same-sex peers could influence the perceived acceptability of sexual practice. In a similar manner, Neufeld and Mate (2005) confirmed that peers substituted the influence of adults and served as the chief source of values, norms, cultures, and behavioral influences such as sexual activities for adolescents. This finding also verifies the results of some previous studies (Haynie, 2002; Maryatun, 2013; Winarni et al., 2016; Srishti and Ayesha, 2018), which confirmed the existence of a relationship between peer pressure with premarital sexual behavior. Peers are considered to exert a major within the field of social transport on adolescent sexual behavior. Peers are one source of data around sex, very noteworthy informing information, states of mind, and sexual behavior of adolescents.

This study found that there was a significant difference in self-esteem between men and women in favor of sex. This contradicts the study of Gentzler and Kerns (2004). Their research work revealed that women having high self-esteem had a greater number of sexual companions than women with low self-esteem. Similarly, a significant difference in exposure to peer pressure between men and women was obtained favoring men. However, contrasting with the findings of the present study, Barnes (2007) advocated that undesirable peer pressure during sexual activity is stronger for women than men.

Another finding of this research was that self-esteem emerged as a unique significant contributor to attitude toward premarital sex which suggests for every unit of increase in self-esteem respondents are 0.63 times less likely to have a favorable attitude toward premarital sex. This partially agrees with the work of Weaver and Herold (2000). In their study, they revealed that 25% of women have practiced unintended sex for the sake of enhancing their lowered self-esteem. On the contrary, 24% of women who did not have sexual intercourse believed that they will improve if they engage in unintentional sexual relationships.

On the contrary, this research result was also consistent with a study conducted by Regan and Dreyer (1999) stated that 24.4% of woman participants in their study experienced premarital sex just to feel “attractive,” which can reflect a facet of self-esteem as the participation in this activity enhance the self-perceived value of the person. Women intentionally perform premarital sex to have good-looking and enhance their self-esteem. Women who had security problems and lived alone want to formulate sexual partnerships (Tambor et al., 1995; Richards, 2013).

In the present study, sex failed to predict attitude toward premarital sex. In contrast, previous studies such as Ojedokun and Balogun (2008) revealed that male students reported more favorable attitudes toward premarital sexual permissiveness than female students. In contrast, according to England and Bearak (2014) women are exposed to a bad judgment than men while it is obviously understood that they committee premarital sex, women expressed a minimum amount in casual sex than men.

Ojedokun and Balogun (2008) reported that besides gender, premarital sexual permissiveness is strongly influenced by living arrangements, with male and female students living alone reporting favorable attitudes toward premarital sexual permissiveness, followed by male and female students living with fellow students and female and male students living with friends. However, male and female students living with guardians and parents reported the least favorable attitudes toward premarital sexual permissiveness. In general, premarital sexual permissiveness is comparable in the level of study and residential area of both male and female students. In a similar manner, the living conditions of teenagers influence their sexual behavior. Adolescents living with friends, families, and boyfriends/girlfriends had a higher probability of starting sexual practices early than their equivalents (Meschke et al., 2000). Research was performed by Tasew (2011) in Ethiopia indicated that premature sexual intercourse was directly related to a deficiency of adequate knowledge and skill on HIV/AIDS, substance use, and alcohol consumption.

## Conclusion and recommendation

Because of negative peer pressure and lack of parental supervision and control, students may take the advantage of individual freedom to practice their sexual wants. The university should open effective guidance and counseling offices and gender-based guidance, especially for first year students.

### Psycho-social interventions

Members of the University community specifically students, teachers, health workers, and parents of students will increase their consciousness about premarital sexual practice. Office of student’s affairs can gate baseline information to design strategies and deliver training that will enable students to enhance their self-esteem and manage negative peer pressures. It can also be a catalyst for the future research works.

## Data availability statement

The original contributions presented in this study are included in the article/supplementary material, further inquiries can be directed to the corresponding author.

## Ethics statement

The studies involving human participants were reviewed and approved by Bahir Dar University College of Educational and Behavioral Science Research and Community Service Dean Office (Nov 10.2020/No EBSRCS12-250). The patients/participants provided their written informed consent to participate in this study.

## Author contributions

The author confirms being the sole contributor of this work and has approved it for publication.

## References

[B1] Abiy T. Teketel E. Aklilu M. (2018). Assessment of pre-marital sex and its associated factors among rift valley university college students, Jimma campus, southwest Ethiopia. *Nurs. Care Open Access J.* 7 27–33.

[B2] Aditomo A. Retnowati S. (2014). Perfectionism, Self-Esteem, and Depression in Teens Final trend. *J. Psychol.* 1 1–14.

[B3] Anyama S. (2019). Relationship between peer pressure, pornography and attitude to premarital sex among adolescents in lagos state. *Int. J. Educ. Res.* 6.

[B4] Barnes K. (2007). The role of mindfulness in romantic relationship satisfaction and responses to relationship stress. *J. Marital. Fam. Ther.* 33 482–500. 10.1111/j.1752-0606.2007.00033.x 17935531

[B5] Berihun H. (2014). *Assessment of the prevalence of premarital sex and unprotected sexual practices among secondary school adolescent students in Sebeta town, Oromia regional state, Ethiopia.* Addis Ababa: Addis Ababa University.

[B6] Bhana A. Lombard C. (2004). Substance abuse, sucidality, and self-esteem in South African adolescents. *J. Drug Educ.* 34 1–17. 10.2190/07C2-P41F-4U2P-JH0Q 15468744

[B7] Bhatta D. Koirala A. Jha H. (2016). Adolescent students attitude towards premarital sex and unwanted pregnancy. *Health Renaiss.* 10 145–149.

[B8] Clasen D. Brown B. (2000). The Multidimensionality of peer pressure in Adolescence. *J. Youth Adolesc.* 14 6–8. 10.1007/BF02139520 24301413

[B9] Cotterell J. (2007). *Social Networks in Youth and Adolescence.* New York, NY: Routledge.

[B10] England B. Bearak H. (2014). The sexual double standard and gender differences in attitudes toward casual sex among U.S. university students. *Demogr. Res.* 30 1327–1338. 10.4054/DemRes.2014.30.46 16041089

[B11] Eysenck S. B. G. Eysenck H. J. (1971). Attitudes to sex, personality and lie scale scores. *Percept. Mot. Skills* 33, 216–218. 10.2466/pms.1971.33.1.216 5095788

[B12] Geckil E. Dundar O. (2011). Turkish adolescent health risk behaviors and self-esteem. *Soc. Behav. Pers.* 39 219–228. 10.2224/sbp.2011.39.2.219

[B13] Gentzler A. L. Kerns K. A. (2004). Associations between insecure attachment and sexual experiences. *Pers. Relationsh.* 11 249–265. 10.1111/j.1475-6811.2004.00081.x

[B14] Gifford S. M. Dodge K. A. Dishion T. J. McCord J. (2005). Peer influence in children and adolescents: Crossing the bridge from developmental to intervention science. *J. Abnorm. Psychol.* 33 255–265. 10.1007/s10802-005-3563-7 15957555 PMC2747364

[B15] Girma B. Assefa D. Tushunie K. (2004). Determinants of condom use among Agaro high school students using behavioral models; Ethiopian. *J. Health Dev.* 18 25–30.

[B16] Habte N. Adu A. Gebeyehu T. Alemayehu S. (2017). Prevalence of premarital sexual practices and its associated factors among high school students in Addis Zemen Town, South Gondar, Ethiopia. *J. Public Health Epidemiol.* 10 356–362.

[B17] Haynie D. (2002). The Relative Nature of Peer Delinquency. *Quant. Criminol.* 2 99–134. 10.1023/A:1015227414929

[B18] Hidayat K. (2013). Effects of Self-Esteem and Moral Reasoning Against Sexual Behavior of Youth Dating in SMK Negeri 5 Samarinda. *J. Psychol.* 12 80–87.

[B19] Ifeoma R. E. (2014). Adolescents’ Attitude towards Premarital Sex. *Mediterr. J. Soc. Sci.* 5 10–12.

[B20] Jones R. (2010). *Happiness at Work, Maximizing Your Psychological Capital for Success.* New York, NY: Wiley Blackwell.

[B21] Lejuez C. W. Simmons B. L. Aklin W. M. Daughters S. B. (2004). Risk-taking propensity and risky sexual behavior of individuals in residential substance use treatment. *Addict. Behav.* 29 1643–1647. 10.1016/j.addbeh.2004.02.035 15451132

[B22] Liu H. C. (2005). *The Effects of Rational-Emotive Group Counseling on Elementary School student’ peer Relations and Rational Belief.* Ph.D thesis, Pingtung city: National Pingtung University of Education.

[B23] Malhotra S. (2008). Impact of the sexual revolution: Consequences of risky sexual behaviors. *J. Am. Phys. Surg.* 13 88–102.

[B24] Manzoni M. L. Lotar M. Ricijas N. (2011). *Peer pressure in adolescence: Boundaries and possibilities*. Saarbrucken: LAP LAMBERT Academic Publishing.

[B25] Maryatun (2013). Role of peers against premarital sexual behavior in teens in SMA Muhammadiyah 3 Surakarta. *Gaster* 10, 39–47.

[B26] Meschke S. Bartholomae R. Zentall W. (2000). Adolescent Sexuality and Parent-Adolescent Processes: Promoting Healthy Teen Choices. *J. Adolesc. Health* 31 264–279. 10.1016/S1054-139X(02)00499-8 12470924

[B27] Mulugeta Y. Berhane Y. (2014). Factors associated with pre-marital sexual debut among unmarried high school female students in Bahir Dar town, Ethiopia: Cross-sectional study. *Reprod. Health* 11:40. 10.1186/1742-4755-11-40 PMC4068072 24885739

[B28] Neufeld S. Mate D. (2005). *Hold on to Your Kids: Why Parents Need to Matter More Than Peers.* New York, NY: Ballantine Books.

[B29] Ojedokun F. Balogun H. (2008). Attitude Towards Littering as a Mediator of the Relationship between Personality Attributes and Responsible Environmental Behavior. *Waste Manag.* 31 2601–2611. 10.1016/j.wasman.2011.08.014 21911286

[B30] Regan P. Dreyer C. (1999). Lust? Love? Status? Young adults’ motives for engaging in casual sex. *J. Psychol. Hum. Sex.* 11 1–24. 10.1300/J056v11n01_01

[B31] Richards N. M. (2013). *Parental care, control, and communication about sex: The relation to risky sexual behaviors and relationship style in emerging adults. Senior Honors Teses. Paper 345. Honors College*. Available online at: https://commons.emich.edu/honors/345

[B32] Robert J. Kuczmarski C. Ogden L. Shumei S. (2002). 2000 CDC Growth Charts for the United States: Methods and development. *Vital Health Stat.* 11. 12043359

[B33] Rosenberg M. (1965). *Society and the adolescent self-image*. Princeton, NJ: Princeton University Press.

[B34] Salih N. Metaferia H. Reda A. Biadgilign S. (2015). Premarital sexual activity among unmarried adolescents in northern Ethiopia: A cross-sectional study. *Sex. Reprod. Health* 6 9–13. 10.1016/j.srhc.2014.06.004 25637418

[B35] Shemsu N. Tilahun B. (2020). Prevalence of premarital sexual practice and associated factors among Goba town high school students. South East-Ethiopia. *J. Clin. Intensive Care Med.* 5 001–006. 10.29328/journal.jcicm.1001027

[B36] Srishti S. Ayesha A. (2018). Attitude towards premarital sex. *Int. J. Adv. Educ. Res.* 3 08–16.

[B37] Tambor E. Terdal S. Downs D. L. (1995). Self-esteem as an interpersonal monitor: The sociometer hypothesis. *J. Pers. Soc. Psychol.* 68 518–530. 10.1037/0022-3514.68.3.518

[B38] Tasew A. (2011). *Sexual Experience and Their Correlates among Jigjiga University Students, Ethiopia.* Ph.D thesis Addis Ababa: Addis Ababa University.

[B39] Tesfaye R. Dereje C. Emiru A. (2016). Premarital Sex in the Last Twelve Months and Its Predictors among Students of Wollega University, Ethiopia. *Ethiop. J. Health Sci.* 26 351–358. 10.4314/ejhs.v26i4.7 27587933 PMC4992775

[B40] Tomas B. Asfew N. Anteneh K. (2017). Prevalence of premarital sexual practice and associated factors among undergraduate health science students of Madawalabu University, Bale Goba, South East Ethiopia: Institution based cross sectional study. *Pan. Afr. Med. J.* 20:209. 10.11604/pamj.2015.20.209.4525 PMC4470411 26113940

[B41] Tsotovor L. A. Dadey G. O. (2021). Gender attitude towards adolescent’s premarital sex in senior and junior high schools in the new Juaben municipality in Ghana. *Innovare J. Educ.* 9 15–21.

[B42] Weaver S. J. Herold E. S. (2000). Casual sex and women: Measurement and motivational issues. *J. Psychol. Hum. Sex.* 12 23–41. 10.1300/J056v12n03_02

[B43] Winarni, Okid P. A. Ruben D. (2016). Association between Self-Esteem, Self-Efficacy, Peers, Parental Controls and Sexual Behavior in Adolescents at High School, Surakarta. *J. Health Promot. Behav.* 1 46–53. 10.26911/thejhpb.2016.01.01.07

[B44] XiayunZuo M. ChaohuaLou M. ErshengGao D. YanCheng P. (2012). Gender Differences in Adolescent Premarital Sexual Permissiveness in Three Asian Cities: Effects of Gender-Role Attitudes. *J. Adolesc. Health* 50 S18–S25. 22340852 10.1016/j.jadohealth.2011.12.001 PMC4235609

[B45] Yamane T. (1967). *Statistics, an introductory analysis* 2nd Edn. New York, NY: Harper and Row.

